# Parallel validation of a green-solvent extraction method and quantitative estimation of multi-mycotoxins in staple cereals using LC-MS/MS

**DOI:** 10.1038/s41598-020-66787-z

**Published:** 2020-06-25

**Authors:** Sefater Gbashi, Patrick Berka Njobeh, Ntakadzeni Edwin Madala, Marthe De Boevre, Victor Kagot, Sarah De Saeger

**Affiliations:** 1 Department of Biotechnology and Food Technology, Faculty of Science, University of Johannesburg, P.O Box 17011, Doornfontein Campus, 2028 Gauteng, South Africa; 2 Department of Biochemistry, School of Mathematical and Natural Sciences, University of Venda, Thohoyandou, South Africa; 3 Centre of Excellence in Mycotoxicology and Public Health, Department of Bioanalysis, Ghent University, 9000 Ghent, Belgium

**Keywords:** Biochemistry, Analytical biochemistry, Mass spectrometry, Environmental sciences

## Abstract

In this study, 15 different mycotoxins were estimated in three staple cereals from selected agro-ecological regions in Nigeria using a ‘novel’ green extraction method, pressurized hot water extraction (PHWE) in comparison to a conventional solvent extraction method. Discrimination of the results of PHWE and solvent extraction using principal component analysis (PCA) and orthogonal projection to latent structures discriminate analysis (OPLS-DA) did not yield any differential clustering patterns. All maize samples (n = 16), 32% (n = 38) of sorghum and 35% (n = 37) of millet samples were positive for at least one of the 15 tested mycotoxins. Contamination levels for the cereals were higher in the warm humid rain forest region and gradually decreased towards the hot and arid region in the north of the country. The results demonstrate the applicability of PHWE as a possible alternative extraction method to conventional methods of extraction, which are solvent based.

## Introduction

Mycotoxins are well-known food and feed contaminants that are produced by ubiquitous toxigenic fungal species belonging mainly to the *Aspergillus*, *Penicillium*, *Fusarium*, *Claviceps* and *Alternaria* genera^1–3^. It has been estimated that approximately 4.5 billion people in the world, of which a majority from sub-Saharan Africa are chronically exposed to uncontrolled amounts of these toxins *via* exposure to contaminated foods^4^. Although about 300 to 400 different mycotoxins have been clearly identified in nature, only a few have received significant research attention due to their economic and health significance^5^, some of which include fumonisins (FBs), aflatoxins (AFs), zearalenone (ZEN) and its analogues, ochratoxins (OTs), T-2 toxin (T-2), and some emerging ones such as alternariol monomethyl ether (AME) and sterigmatocystin (STEG).

Relative to their incessant prevalence in the sub-Saharan African food supply chain, mycotoxins have been implicated in a number of adverse socio-economic effects, ranging from human and animal health, impact on food security, impact on livelihood, damage to the African agricultural export market brand, and impact on Africa’s self-sustainability and increased dependence on foreign aid^5–7^. In the global food market, annual losses associated with mycotoxins have been estimated at approximately one billion metric tons of agricultural produce and food commodities^8,9^. Nigeria, a sub-Saharan African nation, and the most populous country on the continent, is one of the countries that have been severely plagued by the mycotoxin menace in Africa^3,10,11^.

Amidst several factors, favorable environmental conditions for colonization of crops by toxigenic fungal species, coupled with susceptibility of endemic staple crops such as maize, sorghum and millet to mycotoxin proliferation have been identified as a critical precursor to the pervasive impact of the toxins in Nigeria^12–15^. A majority of the Nigerian populace (78 to >85%) rely on these cereal grains for dietary supply of energy, household incomes, as well as food and feed ingredients for their livestock^12,16,17^. The farmers produce these crops under varying agronomic practices and different climatic conditions across the country. Nigeria has a humid tropical climate in its southern region which is close to the equator, and a hot arid climate gradually unfolds towards the northern part of the country, resulting in definable floral and agroecological patterns across the country.

Since these cereal crops are mostly produced by peasant farmers and marketed in local markets within the country and to other neighboring countries *via* unofficial sales channels^18,19^, surveillance of mycotoxin contamination levels seldom occurs^20^. In this regard, despite the compelling evidence of risk exposure to mycotoxins on the Nigerian populace and their effects thereof ^3,21^, there is limited data on mycotoxins in Nigeria. It is thus imperative to routinely monitor the prevalence and levels of these toxins in food/feed in order to adopt appropriate control measures and policies. While it is important to adopt sensitive testing methods for mycotoxins, it is equally expedient to use methods that are fast, effective, more sustainable and environmentally friendly. The present study describes the screening of samples of three staple cereals (maize, sorghum and millet) obtained from selected regions within Nigeria for possible contamination with mycotoxins using a novel green and effective method, pressurized hot water extraction (PHWE) in contrast to a conventional solvent-based extraction followed by HPLC and tandem MS analysis.

## Materials and methods

### Materials

Reference materials, i.e. mycotoxin standards used for the PHWE experiments were purchased from the National Metrological Institute of South Africa (NMISA), and included fumonisin B_1_ (FB_1_), fumonisin B_2_ (FB_2_), fumonisin B_3_ (FB_3_), aflatoxin B_1_ (AFB_1_), aflatoxin B_2_ (AFB_2_), aflatoxin G_1_ (AFG_1_), aflatoxin G_2_ (AFG_2_), alternariol monomethyl ether (AME), sterigmatocystin (STEG), T-2 toxin (T-2), zearalenone (ZEN), α-zearalenol (α-ZEL), β-zearalenol (β-ZEL), ochratoxin A (OTA) and ochratoxin B (OTB). For the solvent extraction, reference standards used included zearalanone (ZAN) and all those mentioned above which were purchased from Biopure (RomerLabs, Tulln, Austria), with the exception of deepoxy-deoxynivalenol (DOM) and AME which were purchased from Sigma-Aldrich (Bornem, Belgium) and FB_3_ supplied by Promec unit (Tygerberg, South Africa).

C18 solid phase extraction (SPE) columns, Biotage Isolute SAX cartridges and MultiSep226 AflaZon+ multifunctional columns were purchased from Alltech (Lokeren, Belgium), Anatech Instruments (Pty) Ltd (South Africa) and Romer Labs (Gernsheim, Germany), respectively. Ten (10) mL NORM-JECT plastic syringe (with Luer lock) and PVDF syringe filters (0.22 µm, with Luer lock) were purchased from Restek (Restek Corporation, Pennsylvania USA). VWR International (VWR International, Leuven, Belgium) supplied the Whatman glass microfiber filters (47 mm diameter, grade GF/A), while Millipore (Bredford, MA, USA) supplied the Ultrafree-MC PVDF centrifugal filters (0.22 µm).

The solvents used for the PHWE experiments included MS-grade acetonitrile, formic acid, dichloromethane (DCM), methanol, iso-octane, ethanol and sulphuric acid which were also purchased from Sigma (South Africa). A Milli-Q Gradient A10 dispensing system (Millipore, Billerica, MA, USA) was used to produce the deionized (ultrapure) water. Sodium sulphate, diatomaceous earth, anhydrous sodium sulphate, potassium chloride, sodium bicarbonate, and dialysis tubing were supplied by Sigma (South Africa). High performance liquid chromatography (HPLC) grade acetonitrile and LC-MS grade methanol, acetonitrile and glacial acetic acid which was used for the solvent extraction were purchased from (Biosolve BV, Valkenswaard, The Netherlands), while Merck (Merck KGaA, Darmstadt, Germany) supplied the ammonium acetate. Nitrogen gas (Air Liquide, Aalter, Belgium) and N-hexane was purchased from BDH Hipersolv Chromanorm (VWR International, Leuven, Belgium). A Milli-QSP Reagent water system (Millipore Corp., Brussels, Belgium) was used to produce the ultrapure water used for this method of extraction (i.e. solvent extraction).

### Methods

#### Sampling and sampling preparations

Sampling. Sampling was done as described by Atehnkeng *et al*.^22^ Sixteen maize (*Zea mays*) samples, 38 sorghum (*Sorghum bicolor*) samples and 37 millet (*Pennisetum glaucum*) samples were obtained from farmers/traders from five different agroecological zones in Nigeria^10,23^ as described in Table 1. Choice of grains was based on consumption patterns of the West African diet, which is characterized by increased consumption of the selected cereals.

**Table 1:** Selected sampling locations for maize, sorghum and millet in Nigeria.

Crop	Number of samples	Agroecological zone	Sub-regions	Cultural/Geopolitical jurisdiction
Maize	16	Humid Rain Forest and Derived Savannah (HRF/DRS)	Lagos, Ondo, Ogun, Osun and Ekiti	Southern Nigeria
Sorghum	12	Humid Rain Forest and Derived Savannah (HRF/DRS)	Ondo, Ogun, Osun and Ekiti	Southern Nigeria
	26	Sahel Savannah (SHS)	Sokoto, Katsina, Kebbi, Jigawa and Kano	Northern Nigeria
Millet	20	Humid Rain Forest and Derived Savannah (HRF/DRS)	Ondo, Ogun, Osun and Ekiti	Southern Nigeria
	9	Northern and Southern Guinea Savannah (NGS/SGS)	Niger, Bauchi, Plateau, Kogi, Benue and Kwara,	North-Central Nigeria
	8	Sahel Savannah (SHS)	Kebbi and Sokoto	Northern Nigeria

The Humid Rain Forest zone (HRF) falls within latitudes 6°4′ & 7°5′N and longitudes 3°5′ & 8°8′E, with maximum temperatures ranging from 26 to 28 °C, and average rainfall between 1,300 and 2,000 mm from two raining seasons in a year. Lying between latitudes 6°8′ & 9°30′N and longitudes 2°40′ & 12°15′E is the Derived Savannah zone (DRS), with maximum temperatures in a year averaging 25 to 35 °C and a bimodal rainfall averaging between 1,300 mm and 1,500 mm annually. The Southern Guinea Savannah (SGS) is characterized by a mean bimodal rainfall distribution between 1,000 to 1,300 mm per annum, maximum temperatures averaging 26 to 38 °C, and lies within latitudes 8°4′ & 11°3′N and longitudes 2°41′ & 13°33′E, while the Northern Guinea Savannah (NGS) is characterized by a single raining season per annum averaging between 900 to 1000 mm, and maximum temperatures ranging between 28 and 40 °C, and lies within latitudes 9°10′ & 11°59′N and longitudes 3°19′ & 13°37′E. The Sahel Savannah (SHS) lies within latitudes 12°2′ & 13°8′N and longitudes 3°9′ & 13°9′E, and is characterized by a Saharan climate, with maximum temperatures averaging between 30 to 40 °C and a single raining season per annum with rainfall distribution averaging between 650 and 1,000 mm^22,23^.

Pressurized hot water extraction (PHWE). The cereal samples (maize, sorghum and millet) were milled to sieve size of <0.6 to 1 mm using a mechanical blender. Extraction was performed using a laboratory-scale PHWE equipment^24^ operated at previously optimized extraction conditions of 55/45 (water/ethanol v/v) solvent composition and 162 °C temperature^25^. For the extraction, 4 g of grounded cereal sample was thoroughly mixed with 3 g of diatomaceous earth and transferred into the extraction cell (70 × 30 mm and approximately 20 mL) which was contained in a (gas chromatography) GC oven (Carlo Erba Instruments, Italy) maintained at a temperature of 162 °C using a digital temperature controllable unit (±1 °C). The extraction solvent [55/45 (water/ethanol v/v)] was pumped at a constant flow rate of 5 mL/min through the extraction cell *via* a stainless-steel tubbing (1.58 mm in outer dimension and 0.18 mm inner dimension), and the pressure maintained at 1000 ± 200 psi by means of a back-pressure valve (Swagelok, Johannesburg, South Africa). The extract was made to pass through a cooling coil and collected into a 50 mL centrifuge tube up to the 50 mL mark. Two (2) mL of the extracts were filtered through a 0.22 µm PVDF syringe filter into a 2 mL HPLC vial for subsequent analysis on HPLC-MS/MS.

Solvent extraction. Solvent extraction was performed using the method of Majeed *et al*.^26^. Briefly, 5 g of samples were spiked with internal standards, DOM (1 µg/kg) and ZAN (1 µg/kg), prior to extraction. For the extraction, 20 mL of extraction solvent, acetonitrile/water/acetic acid (79/20/1, v/v/v), added to the spiked samples, agitated for 1 h on an overhead shaker (AG 6 A, Exacta, Mery sour Oise, France) and centrifuged for 15 min at 3,300 g using an IEC Central (type MP4) centrifuge (VWR International, Leuven Belgium). The supernatant was passed through a pre-conditioned (10 mL of the extraction solvent) octadecyl (C18) solid phase extraction (SPE) column (Grace octadecyl C18, Lokeren, Belgium) under gravity. A second extraction was performed on similar samples by adding 5 mL of extraction solvent, agitating, centrifuging and passing through the SPE column as described above. The total volume of the eluate was adjusted to 25 mL and defatted with 10 mL n-hexane. The defatted extract was split into two equal parts for further clean-up using different approaches. The first portion (10 mL defatted extract) was diluted with 20 mL acetonitrile/acetic acid (99/1, v/v) and was subjected to clean-up by a Multisep226, Afla-ZON + multifunctional column, under gravity, followed by further column washing using 5 mL of acetonitrile/acetic acid (99/1, v/v). The second portion (10 mL defatted extract) was filtered using a Whatman glass microfilter (VWR International, Zaventem, Belgium). All of the first extract was combined with 2 mL of the second extract, evaporated to dryness under a stream of nitrogen gas at 40 °C. The residue was reconstituted in 150 µL of mobile phase, methanol/water/acetic acid (57.20/41.80/1, v/v/v) and 5 mM ammonium acetate, and filtered through a 0.22 µm Ultrafree-MC centrifugal filter (Bedford, MA, USA) at 14,000 g for 5 min.

#### Liquid chromatography coupled to tandem mass spectrometry (LC-MS/MS)

Chromatographic separation and HPLC-MS/MS for PHWE. Chromatographic separation, detection and quantitation of mycotoxin levels was achieved using Shimadzu HPLC-MS/MS 8030 equipment (Shimadzu Corporation, Tokyo, Japan). The system consisted of a chromatograph (LC-30AD Nexera) linked to an autosampler (SIL-30 AC Nexera). Two µL of sample were injected and pumped through a Raptor ARC-18 column (2.7 µm, 2.1 mm × 100 mm) (Restek Corporation, Pennsylvania USA) maintained at 40 °C in a column oven (CTO-20 AC Prominence). A binary pump (LC-20AD) connected to the system was used to pump the mobile phases A (aqueous phase) and B (organic phase) through the column at a constant flow rate of 0.2 mL/min. Mobile phase A consisted of 0.1% formic acid (FA) in deionized water, and mobile phase B consisted of 0.1% FA in methanol/acetonitrile (50/50 v/v). The elution gradient program started with pumping 10% B for 0.1 min, which was steadily increased to 95% B within 8.4 min, held constant at 95% B for 3 min, initial gradient condition of 10% B re-established within 1 min, and then the column was allowed to re-equilibrate at this condition for 4.5 min prior to the next injection, making a total run time of 17 min.

Separated analytes were delivered to a Shimadzu triple-quadrupole MS model 8030 (Shimadzu Corporation, Kyoto, Japan) instrument equipped with an electron spray ionization operated in positive mode (ESI^+^). A time-scheduled ultrafast multiple reaction monitoring (MRM) MS method was used for the identification and quantification of the analytes of interest. To improve specificity and confidence in analytical detection, two MRM-transitions were monitored per analyte. The desolvation line (DL) temperature was 250 °C, heat block temperature was 400 °C, drying gas flow rate was 15 L/min, and interface nebulizing gas flow rate was 3 L/min. The Shimadzu LabSolutions software was used for subsequent data visualization and analysis. The optimized chromatographic and MS method parameters of the 15 mycotoxins under investigation are presented in Table 2.

**Table 2:** MRM transitions, optimized MS conditions and retention times of mycotoxins.

S/No	Mycotoxin	Ret. time (min)	Precursor (*mz*)	Products (*mz*)	Q1 Pre bias (V)	CE (eV)	Q3 Pre bias (V)
1.	AFB_1_	8.25	313.00	241.00*	−22.00	−41.00	−23.00
				285.10	−22.00	−24.00	−29.00
2.	AFB_2_	8.01	315.00	259.10*	−22.00	−31.00	−25.00
				287.00	−23.00	−26.00	−30.00
3.	AFG_1_	7.77	329.00	243.00*	−12.00	−28.00	−23.00
				311.10	−16.00	−24.00	−14.00
4.	AFG_2_	7.17	331.00	245.10*	−12.00	−32.00	−24.00
				313.00	−12.00	−24.00	−20.00
5.	AME	10.13	273.00	128.10*	−10.00	−49.00	−21.00
				115.05	−18.00	−54.00	−19.00
6.	FB_1_	7.97	722.20	352.20*	−34.00	−42.00	−11.00
				334.30	−20.00	−42.00	−11.00
7.	FB_2_	8.95	706.10	336.30*	−20.00	−38.00	−22.00
				318.30	−26.00	−41.00	−22.00
8.	FB_3_	8.75	706.30	336.30*	−40.00	−39.00	−11.00
				354.40	−20.00	−35.00	−24.00
9.	OTA	10.13	403.80	239.00*	−15.00	−27.00	−24.00
				221.00	−12.00	−38.00	−21.00
10.	OTB	9.33	370.10	205.00*	−13.00	−22.00	−21.00
				324.10	−13.00	−14.00	−22.00
11.	STEG	10.45	324.90	310.00*	−22.00	−24.00	−30.00
				281.10	−22.00	−40.00	−27.00
12.	T-2	9.67	467.20	245.10*	−13.00	−11.00	−16.00
				305.20	−22.00	−11.00	−20.00
13.	ZEN	10.06	319.10	185.00*	−12.00	−27.00	−30.00
				187.10	−15.00	−21.00	−19.00
14.	α-ZEL	9.42	323.10	277.20*	−17.00	−17.00	−18.00
				305.20	−24.00	−9.000	−20.00
15.	β-ZEL	8.95	323.10	277.20*	−16.00	−16.00	−18.00
				305.20	−16.00	−11.00	−20.00

Key: S/No: serial number. Ret. Time: retention time. Q1 Pre bias: quadruple one pre-rod bias. Q3 Pre bias: quadruple three pre-rod bias. CE: collision energy. *Quantitative product ion. AFB_1_: aflatoxin B_1_, AFB_2_: aflatoxin B_2_. AFG_1_: aflatoxin G_1_. AFG_2_: aflatoxin G_2_. AME: alternariol monomethyl ether. FB_1_: fumonisin B_1_. FB_2_: fumonisin B_2_. FB_3_: fumonisin B_3_. OTA: ochratoxin A. OTB: ochratoxin B. STEG: sterigmatocystin. T-2: T-2 toxin. ZEN: zearalenone. α-ZEL: α-zearalenol. β-ZEL: β-zearalenol.

Chromatographic separation and LC-MS/MS for solvent extraction. A Waters Acquity UPLC system (Waters, Milford, MA, USA) linked to a Waters Micromass Quattro Micro triple-quadrupole mass spectrometer (Waters, Milford, MA, USA) was used for chromatographic separation, detection and quantification of the extracts obtained from the solvent extraction. The above described LC-MS/MS system was equipped with a Waters Symmetry C18 analytical column (5 µm, 2.1 × 150 mm) and a Waters Sentry guard column (3.5 µm 2.1 × 10 mm) purchased from the same vendor. The column oven was kept at room temperature (25 °C), and 20 µL of sample was injected into the column. The aqueous portion of the mobile phase (*i.e*., mobile phase A) consisted of water/methanol/acetic acid (94/5/1, v/v/v) and 5 mM ammonium acetate, while the organic portion of the mobile phase (*i.e*., mobile phase B) consisted of methanol/water/acetic acid (97/2/1, v/v/v) and 5 mM, ammonium acetate. The gradient elution program and MS parameters are as described by Monbaliu *et al*.^27^.

#### Validation of the modified PHWE method for multi-mycotoxin extraction

The effects of the cereal-matrix components on the analytical signals of the different mycotoxins in the MS were determined using the signal suppression/enhancement method described by Arroyo-Manzanares *et al*.^28^ and Sulyok *et al*.^29^. Calibration curves were plotted for standards prepared in analyte-free sample extracts, as well as for standards prepared in neat organic solvents (100% methanol). Matrix-effect (ME) was determined as the percentage ratio of the difference between the slope of matrix-matched calibration curve and neat standards calibration curve divided by the slope of neat standards calibration curve (Eq. 1).

1 $$ME=\frac{Slop{e}_{m}-Slop{e}_{n}}{Slop{e}_{n}}\times 100$$

where *ME* is the matrix effect, *Slope*_*m*_ is the slope of calibration curve of standards prepared in sample extracts and *Slope*_*n*_ is the slope of standards prepared in neat solvent.

The limits of detection (LOD) and limits of quantification (LOQ) (Eq. 2) of the mycotoxins were determined using the signal-to-noise ratio of the matrix-matched standards as described by Kim *et al*.^30^. Linearity was determined by least-square regression of a 6-point matrix-matched calibration curve within the ranges of 9 to 5,000 µg/kg depending on the mycotoxin. Recovery efficiency of the method was determined by spiking analyte-free samples with known concentrations of mycotoxins, extracting the analytes the same day using PHWE as described above. The percentage ratio of post-extraction concentration (recovered concentration) to that of pre-extraction concentration (initial concentration) was taken as the recovery value (Eq. 3)^28^.

2 $$Limi{t}_{DQ}=F\times \left[\frac{C}{\left(\frac{S}{N}\right)}\right]$$

3 $$Recovery( \% )=\frac{{E}_{r}}{{E}_{i}}\times 100$$

where *Limit*_*DQ*_ is the *LOD* or *LOQ* depending on the value of the multiplication factor *F*, which is 3.33 for *LOD* and 10 for *LOQ*. *C* is the concentration, while *S* is the signal at concentration *C*, and *N* is the noise level at similar concentration. *E*_*r*_ is the recovered concentration after spiking, and *E*_*i*_ is the spiked concentration.

#### Multivariate discriminant analysis

The pre-processed data set was subjected to multivariate discriminant analysis in order to scrutinize for discriminatory patterns between the two extraction methods. Using the SIMCA-P^+^ 14.0 chemometrics software (Umetrics, MKS Instruments Inc., Sweden), the data was mean-centered, Pareto-scaled^31^ and subjected to PCA and OPLSD-DA analysis in order to extract maximum information from the data set. The adopted models, PCA and OPLS-DA, are advanced dimensionality reduction tools which highlights contrasts or similarities between data groups *via* construction of few interpretable linearly uncorrelated variables called latent variable or principal components from the dataset^32^. PCA does not supervise the construction of latent variables from the dataset while the OPLS-DA supervises the construction of latent variables from the dataset^31,32^.

For the OPLS-DA model, the data variables were classified into two major groups depending on the adopted method of analysis, either PHWE or solvent extraction. This was critical because, OPLS-DA is a supervised model, as such, information regarding variable class member is a prerequisite for location of the principal components. Usually, OPLS-DA is best applicable when there are only two classification groups in the data set, such as a control group and a dependent group. As such, classification of the data set into two groups permitted the extraction of a between-class variation referred to as the Y-predictive block, and a within-class variation referred to as the Y-orthogonal block or uncorrelated variation^33^. By doing so, OPLS-DA maximizes the discrimination of the two groups of variables and provides an improved model interpretability without modifying its predictive power^34^.

For the evaluation of model performances, the quantitative *goodness-of-fit* parameters *i.e., R2*X(cum) and *Q2X*(cum) values, and the *goodness-of-prediction* parameters *i.e*., the *Q2*(cum) values, were calculated. The *R2*X(cum) and *Q2X*(cum) values for the PCA model were used to measure the degree to which the latent structures (*i.e*., principal components) describe the variations and patterns in the data set^32^. Whereas for the OPLS-DA model, the *R2*X(cum) was used to estimate the cumulative fraction of the variation of the *X* variables explained by the model, *R2*Y(cum) was used to measure the cumulative ratio of the variation of the *Y* variables explained by the model, and the *Q2*(cum) estimated the cumulative predictive capacity of the full model^31,35^. For internal validation of the OPLS-DA models to assess the statistical significance of the model, the goodness-of-fit and goodness-of-prediction of the OPLS-DA model was compared with those of 100 random Y-permutated models, which generates a distribution of *Q2* values that are suitable for testing the null hypothesis for a model’s *Q2*^32,36^.

## Results and discussion

Pressurized hot water extraction, a ‘novel’ green extraction technique was adopted for the analysis of multi-mycotoxin in 91 samples of maize, sorghum and millet intended for human consumption obtained from different agroecological zones in Nigeria as described in Section 2.2.1. In order to authenticate the PHWE method, a method validation was performed, as well as, a comparison with a solvent-based extraction method.

### Method validation and comparative evaluation of PHWE and solvent extraction

The results of PHWE method validation in comparison with solvent extraction is presented in Appendix A and Table 3. In order to compensate for matrix effects, matrix-matched calibrations were adopted for quantification of the mycotoxin concentrations in the samples for the two methods.

**Table 3:** Method validation parameters for PHWE and solvent extraction^#^ of multi-mycotoxin in maize, sorghum and millet matrices.

Mycotoxin	Maize				Sorghum				Millet			
	LOD (µg/kg)	LOQ (µg/kg)	Recovery (%)	RSDr (%)	LOD (µg/kg)	LOQ (µg/kg)	Recovery (%)	RSDr (%)	LOD (µg/kg)	LOQ (µg/kg)	Recovery (%)	RSDr (%)
AFB_1_	0.94	2.8	108	3.1	0.37	1.1	104	1.7	2.2	6.6	97	10
	0.28^#^	0.9^#^	100^#^	1.6^#^	0.89^#^	2.7^#^	101^#^	6.4^#^	2.0^#^	6.0^#^	101^#^	5.3^#^
AFB_2_	0.08	0.25	98	12	0.07	0.20	108	3.2	1.0	3.1	72	9.0
	0.04^#^	0.13^#^	100^#^	0^#^	0.76^#^	2.3^#^	101^#^	11^#^	3.4^#^	10^#^	101^#^	8.0^#^
AFG_1_	0.43	1.3	126	13	1.2	3.7	125	4.3	2.7	8.2	96	8.3
	0.13^#^	0.38^#^	100^#^	0.83^#^	0.27^#^	0.80^#^	101^#^	9.6^#^	1.0^#^	2.9^#^	100^#^	4.1^#^
AFG_2_	1.2	3.6	107	8.2	1.2	3.7	115	2.6	2.7	8.1	85	6.5
	4.2^#^	13^#^	100^#^	0.85^#^	1.7^#^	4.9^#^	102^#^	11^#^	7.3^#^	22^#^	100^#^	6.4^#^
AME	2.4	7.2	77	11	5.7	17	79	11	26	78	72	14
	0.86^#^	2.6^#^	100^#^	2.9^#^	3.9^#^	12^#^	101^#^	5.3^#^	6.8^#^	21^#^	101^#^	7.8^#^
FB_1_	2.3	6.8	111	9.6	1.1	3.2	81	2.9	2.2	6.5	133	6.8
	1.3^#^	3.8^#^	100^#^	2.7^#^	1.3^#^	3.9^#^	101^#^	8.6^#^	5.1^#^	15^#^	102^#^	9.4^#^
FB_2_	4.3	13	89	1.7	7.3	22	78	5.2	0	0.01	119	11
	3.3^#^	9.8^#^	100^#^	1.8^#^	4.7^#^	14^#^	101^#^	7.0^#^	5.6^#^	17^#^	102^#^	11^#^
FB_3_	0.06	0.18	95	5.5	0.07	0.20	84	6.3	0.0	0.01	72	8.3
	2.7^#^	8.2^#^	100^#^	0.77^#^	0.63^#^	1.9^#^	101^#^	8.0^#^	1.3^#^	4.0^#^	102^#^	14^#^
OTA	0.44	1.3	75	8.8	0.17	0.50	72	8.9	0.40	1.2	67	12
	0.32^#^	0.95^#^	100^#^	1.4^#^	1.1^#^	3.3^#^	101^#^	7.8^#^	0.54^#^	1.6^#^	100^#^	8.3^#^
OTB	0.11	0.32	77	6.3	0.07	0.21	79	4.9	0.18	0.55	72	7.9
	ND^#^	ND^#^	ND^#^	ND^#^	ND^#^	ND^#^	ND^#^	ND^#^	ND^#^	ND^#^	ND^#^	ND^#^
STEG	0.50	1.5	95	8.7	0.33	0.99	90	3.2	1.6	4.7	92	13
	0.07^#^	0.21^#^	100^#^	1.6^#^	0.68^#^	2.1^#^	101^#^	5.4^#^	1.4^#^	4.3^#^	100^#^	3.1^#^
T-2	0.04	0.13	87	16	0.07	0.20	84	6.2	1.1	3.4	69	12
	0.86^#^	2.6^#^	100^#^	1.1^#^	2.5^#^	7.4^#^	101^#^	6.6^#^	2.6^#^	7.7^#^	100^#^	5.8^#^
ZEN	1.6	4.9	80	9.8	0.88	2.6	93	3.4	20	59	83	9.5
	4.2^#^	13^#^	99^#^	4.7^#^	15^#^	44^#^	102^#^	20^#^	16^#^	47^#^	102^#^	15^#^
α-ZEL	41	123	84	19	60	180	73	5.3	26	79	100	13
	ND^#^	ND^#^	ND^#^	ND^#^	ND^#^	ND^#^	ND^#^	ND^#^	ND^#^	ND^#^	ND^#^	ND^#^
β-ZEL	12	36	74	7.4	17	50	83	4.1	14	42	73	12
	ND^#^	ND^#^	ND^#^	ND^#^	ND^#^	ND^#^	ND^#^	ND^#^	ND^#^	ND^#^	ND^#^	ND^#^
Mean	1.2	3.6	96	8.9	1.5	4.6	93	4.9	5.0	15	88	10
	1.5^#^	4.6^#^	100^#^	1.7^#^	2.7^#^	8.2^#^	101^#^	8.8^#^	4.4^#^	13^#^	101^#^	8.1^#^
Sig. (2-tailed)	0.59	0.58	0.34	0	0.38	0.38	0.12	0.01	0.83	0.83	0.06	0.15

Key: ^#^Parameter values for solvent extraction. ND: not determined. Recovery values are presented mean of duplicate determinations ± standard deviation of mean. LOD: Limit of detection; LOQ: Limit of quantification. AFB_1_: aflatoxin B_1_. AFB_2_: aflatoxin B_2_. AFG_1_: aflatoxin G_1_. AFG_2_: aflatoxin G_2_. AME: alternariol monomethyl ether. FB_1_: fumonisin B_1_. FB_2_: fumonisin B_2_. FB_3_: fumonisin B_3_. OTA: ochratoxin A. OTB: ochratoxin B. STEG: sterigmatocystin. T-2: T-2 toxin. ZEN: zearalenone. α-ZEL: α-zearalenol. β-ZEL: β-zearalenol.

#### Validation of PHWE and solvent extraction

The performances of both methods showed good consistency with EC, AOAC, and ICH guidelines^28,37–40^. The linearity correlation (*R*^2^) of PHWE ranged from 0.98 to 1.00 for the 15 mycotoxins in all 3 sample matrices within the linear ranges of 60 to 2,000 µg/kg for FB_1_, 16 to 500 µg/kg for FB_2_, 10 to 300 µg/kg for AFB_1_ and 30 to 1,000 µg/kg for the other analytes (Appendix A). The benchmark for the acceptance of linearity of *R*^2^ equal or higher than 0.95 by the International Conference on Harmonization (ICH) was fulfilled for all understudied analytes^39^, though there existed a significant difference (p ≤ 0.05) in the linearities of the two methods. The sensitivity of the methods was determined by assessing the LOD’s and LOQ’s of the method for each of the mycotoxins. For PHWE, the LOD’s and LOQ’s ranged respectively from 0.06 to 41 µg/kg and 0.32 to 123 µg/kg for maize, 0.07 to 98 µg/kg and 0.21 to 295 µg/kg for sorghum, and 0 to 26 µg/kg and 0.01 to 79 µg/kg for millet (Table 3). These values were sufficiently low for detection and quantitation of small amounts of the analytes under investigation in cereal grains^12,30^, as such, trace amounts of the analytes in the sample extracts can be quantitatively reported with a high degree of confidence. There was no statistically significant difference (p ≤ 0.05) between the LOD’s and LOQ’s of PHWE and the solvent extraction method.

The recovery rates of the 15 mycotoxins varied from 74 to 126% in maize, 73 to 115% in sorghum and 67 to 133% in millet for PHWE, which is not far from the AOAC recommendations of between 60% to 125% for foodstuff contaminated with 10 μg/kg of mycotoxins, and the EC recommendations of 60 and 130%^28,40^. The recovery rates for the solvent extraction method varied from 99 to 100% for maize, 100 to 101% in sorghum, and 100 to 102% for millet. Though the recoveries of the solvent extraction method were more consistent and closer to 100%, when compared to those of PHWE using the Independent Sample’s T-test at a 95% probability, there existed no statistically significant differences in the mean recoveries of the two methods. The RSDr values for all the analytes in maize, sorghum and millet matrices ranged from 2 to 19% for PHWE and satisfied the guideline criterion of <25% by CODEX and the EC^28,38,40^. In comparison with the RSD values for the solvent extraction, those of maize ranged from 0 to 5% and 5 to 20% for sorghum. These values were significantly different (p ≤ 0.05) from the corresponding RSD values for PHWE while those of millet (3 to 15%) were not significantly different (p > 0.05).

In general, there was less variability in the validation parameters (*i.e*., recovery, linearity and RSD) of the solvent extraction method as compared to those of PHWE. This could be due to the fact that the solvent extraction method involved multiple clean-up steps (defatting and two SPE purification procedures using different SPE cartridges). This was in addition to the use of two internal standards to correct for loss of analytes during the sample preparation or injection steps in the analytical process. On the other hand, PHWE was designed with the aim of reduction of cost and the amount of harmful organic solvents used during extraction and increased speed of the analytical process. Forfeiture of a clean-up step facilitated the achievement of these objectives. This however contributed to the higher variations observed in the PHWE analytical results, which was not unexpected. Such variations are negligible provided they were within the acceptable limits stipulated by regulatory bodies^28,38,40–43^. As would be discussed in the succeeding sections of this chapter, the overall variations between the two analytical procedures have been shown to be statistically insignificant.

#### Comparative evaluation of PHWE and solvent extraction

Principal component analysis (PCA) and orthogonal projection to latent structures discriminate analysis (OPLS-DA) approaches were adopted to scrutinize the entire data set (*i.e*., combined data from method validation and samples analysis) for inherent global discriminatory patterns such as multiple pairwise correlations and/or co-variances between the data obtained by the two extraction methods, which may not be readily observed by using conventional statistical analysis.

**Figure 1:**
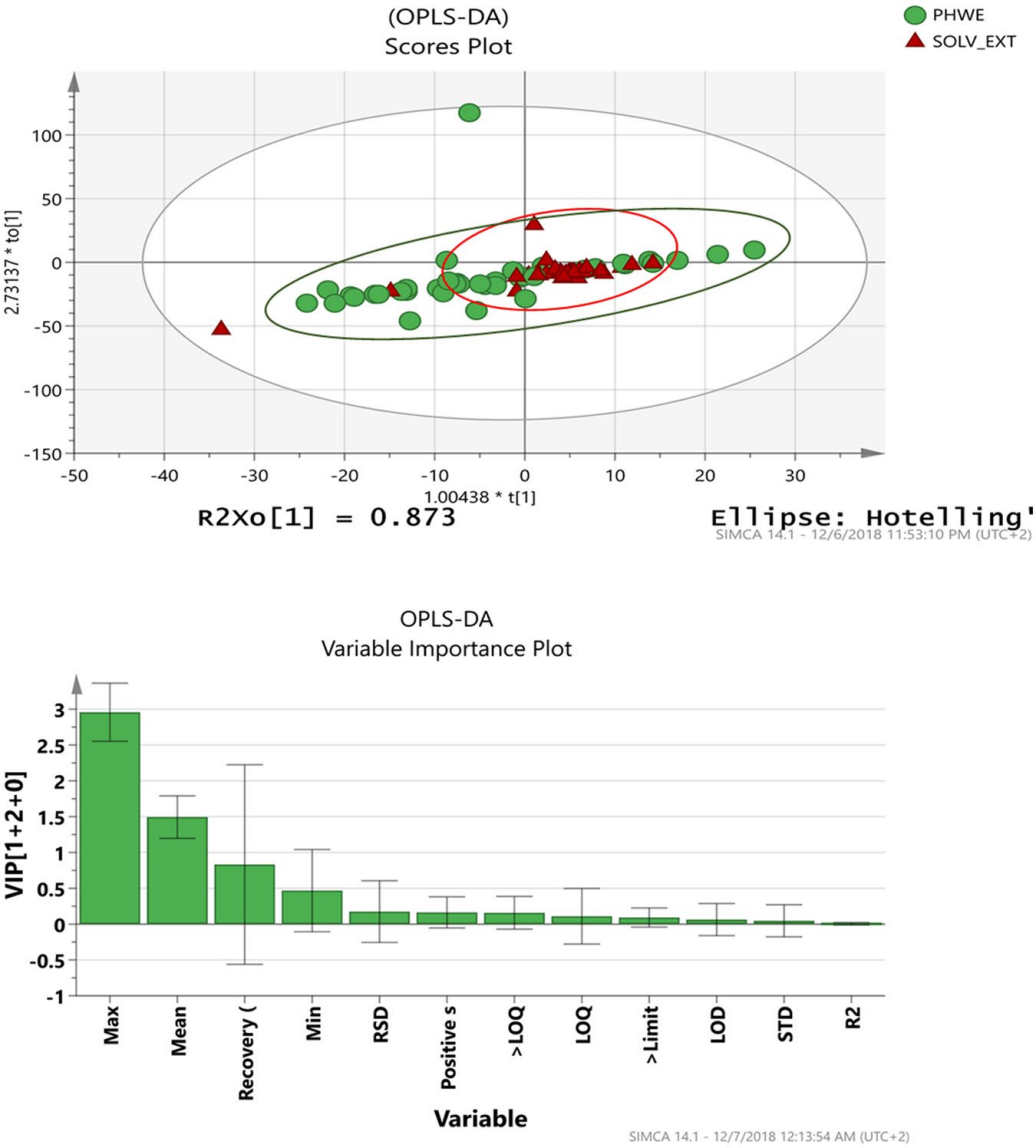
OPLS-DA scores plot and variable importance plot (VIP) plot for discriminate analysis of PHWE and solvent extraction.

Discriminatory analysis of PHWE and solvent extraction methods using PCA and OPLS-DA. The results of the PCA and OPLS-DA analysis of the data and their corresponding model-fit quality parameters are shown in Table 4 and Fig. 1. PCA was used for an initial screening of the overall structure of the data set for discriminatory patterns and detection of outliers. The results revealed a single latent variable, which indicated there were no differential patterns between the variables pertaining to PWHE and corresponding variables pertaining to the solvent extraction. Notwithstanding, the PCA model accounted for 97% of the variations in the data set [*i.e., R2*X(cum) = 0.97], with a predictive ability of 77% [*i.e., Q2X*(cum) = 0.77]. This observation was in agreement with the results of the Independent samples t-test performed on each of the variables from the two extraction methods (Appendix A), where it was observed that the majority of the variables were not significantly different (p > 0.05).

**Table 4:** Summary of PCA-X model and OPLS-DA models with the corresponding model validation parameters.

Model	Data classification	Number of observations	Number of components	*R2*X(cum)	*R2*Y(cum)	Q2(cum)
PCA	None	84 (*X* = 14, *Y* = 2)	1	0.97	—	0.77
OPLS-DA	PHWE & SOLV-EXT	84 (*X* = 14, *Y* = 2)	1 predictive and 2 orthogonal	0.99	−0.11	0.05

Key: PCA-principal component analysis. OPLS-DA-orthogonal projection to latent structures discriminate analysis. PHWE-pressurized hot water extraction. SOLV-EXT-solvent extraction.

Since PCA does not take into account the classification label associated with the data set, discrimination of the data groups is not maximized. This problem is solved by the OPLS-DA model which supervises the construction of the latent variables, hence, yielding a more class-specific discrimination of the data. Results of the OPLS-DA analysis showed the construction of 2 explanatory and 1 predictive principal component with a 99% total explained variation in *X* [*i.e., R2*X(cum) = 0.99]. However, the model could not account for the cumulative variations in Y [*R2*Y(cum) = −0.11], and the total amount of predicted variability in the full model was low [*i.e., Q2*(cum) = 0.05]. As such, despite the OPLS-DA models making reference to the pre-defined sample class membership in order to maximize separation of the data, there was no clear separation of the data groups. A pattern which can be more clearly visualized on the OPLS-DA scores plot (Fig. 1). On this score plot, the green circular dots represent variables of the PHWE class, while the red triangular dots represent variables of the solvent extraction (*i.e*., SOLV-EXT) class. Variables from the two data groups can be seen clustered together towards the middle of the plot and while some arbitrarily distributed across the graph. This is an indication of intrinsic similarities and randomness in the patterns within and between the groups.

This observation is in strong agreement to the results of PCA analysis as well as the Independent Sample’s T-test. Overall, PHWE compared well with the solvent-based extraction method, and other validated methods for multi-mycotoxin extraction reported in literature^28–30,44^. Hence, considering its good validation performance, it was deemed adequate for investigating the natural occurrence of mycotoxins in food commodities.

### Mycotoxin contamination of staple cereals

The above validated PHWE method together with the solvent extraction method was used for the screening of 15 different mycotoxin in samples of maize (n = 16), sorghum (n = 38) and millet (n = 37) obtained from six agroecological zones from Nigeria (Table 5). Only samples contaminated with mycotoxins at levels above the respective LODs were considered positive. The minor differences between the results from the two extraction methods, are possibly due to the differences in the LOD’s and LOQ’s, as well as the recovery rates of the two methods. For the sake of discussing the results, in the remaining sections of this paper, reference is made only to the results of the PHWE.

**Table 5:** Mycotoxin contamination of maize, sorghum and millet from Nigeria using PHWE and solvent extraction^#^.

Mycotoxin	Maize (n = 16)					Sorghum (n = 38)					Millet (n = 37)				
	Positive samples (%)	Mean* (µg/kg)	Range (µg/kg)	Samples > LOQ (%)	Samples > Limit (%)	Positive samples (%)	Mean* (µg/kg)	Range (µg/kg)	Samples > LOQ (%)	Samples > Limit (%)	Positive samples (%)	Mean* (µg/kg)	Range (µg/kg)	Samples > LOQ (%)	Samples > Limit (%)
*AFB_1_	69	54	6.5–315	69	69	8	3.6	7.3–116	8	8	5	2.4	38–50	5	5
	44^#^	18^#^	15–74^#^	44^#^	44^#^	0^#^	0^#^	<LOQ^#^	0^#^	0^#^	3^#^	0.33^#^	<LOQ-9.4^#^	3^#^	3^#^
AFB_2_	69	1.3	0.31–7.8	63	0	0	0^#^	<LOQ	0	0	0	0	<LOQ	0	0
	44^#^	7.0^#^	3.3–13^#^	44^#^	0^#^	0^#^	0^#^	<LOQ^#^	0^#^	0^#^	0^#^	0^#^	<LOQ^#^	0^#^	0^#^
AFG_1_	0	0	<LOQ	0	0	3	0.19	<LOQ-7.3	3	0	0	0	<LOQ	0	0
	0^#^	0^#^	<LOQ^#^	0^#^	0^#^	0^#^	0^#^	<LOQ^#^	0^#^	0^#^	0^#^	0^#^	<LOQ^#^	0^#^	0^#^
AFG_2_	0	0	<LOQ	0	0	0	0	<LOQ	0	0	0	0	<LOQ	0	0
	0^#^	0^#^	<LOQ^#^	0^#^	0^#^	0^#^	0^#^	<LOQ^#^	0^#^	0^#^	0^#^	0^#^	<LOQ^#^	0^#^	0^#^
*ΣAFs	69	56	16–323	50	69	8	3.8	15–116	8	8	5	2.4	38–50	5	5
	44^#^	21^#^	18–83^#^	44^#^	44^#^	0^#^	0^#^	<LOQ^#^	0^#^	0^#^	3^#^	0.67^#^	<LOQ^#^	0^#^	3^#^
AME	0	0	<LOQ	0	0	3	0.99	<LOQ-38	3	0	8	21	128–343	8	0
	0^#^	0^#^	<LOQ^#^	0^#^	0^#^	3^#^	0.89^#^	<LOQ-34^#^	3^#^	0^#^	16^#^	8.5^#^	24–117^#^	16^#^	0^#^
*FB_1_	100	2,033	17–7,947	100	0	8	17	188–248	8	0	0	0	<LOQ	0	0
	94^#^	1,644^#^	68–7,105^#^	94^#^	0^#^	5^#^	6.8^#^	97–159^#^	5^#^	0^#^	0^#^	0^#^	<LOQ^#^	0^#^	0^#^
FB_2_	100	1,525	37–5,961	94	6	0	0	<LOQ	0	0	0	0	<LOQ	0	0
	88^#^	527^#^	57–2,074^#^	88^#^	0^#^	0^#^	0^#^	<LOQ^#^	0^#^	0^#^	0^#^	0^#^	<LOQ^#^	0^#^	0^#^
FB_3_	94	292	7.5–767	94	0	3	0.41	<LOQ -16	3	0	0	0	<LOQ	0	0
	81^#^	186^#^	33–689^#^	81^#^	0^#^	0^#^	0^#^	<LOQ^#^	0^#^	0^#^	0^#^	0^#^	<LOQ^#^	0^#^	0^#^
*ΣFB_1_&B_2_	100	3,851	30–1,460	100	44	8	17	188–248	8	0	0	0	<LOQ	0	0
	94^#^	2,171^#^	68–9,179^#^	94^#^	19^#^	5^#^	6.8^#^	97–159^#^	5^#^	0^#^	0^#^	0^#^	<LOQ^#^	0^#^	0^#^
*OTA	31	8.3	6.5–54	31	31	5	0.59	7.5–15	5	5	5	0.73	6.9–20	5	5
	13^#^	4.0^#^	4.7–54^#^	13^#^	6^#^	0^#^	0^#^	<LOQ^#^	0^#^	0^#^	0^#^	0^#^	<LOQ^#^	0^#^	0^#^
OTB	13	1.5	7.8–17	13	0	3	0.20	<LOQ -7.5	3	0	5	0.37	6.8–6.9	5	0
	ND^#^	ND^#^	ND^#^	ND^#^	ND^#^	ND^#^	ND^#^	ND^#^	ND^#^	ND^#^	ND^#^	ND^#^	ND^#^	ND^#^	ND^#^
ΣOTs	31	9.8	6.5–58	31	0	5	0.79	7.5–23	5	0	5	1.1	14–27	5	0
	ND^#^	ND^#^	ND^#^	ND^#^	ND^#^	ND^#^	ND^#^	ND^#^	ND^#^	ND^#^	ND^#^	ND^#^	ND^#^	ND^#^	ND^#^
STEG	13	0.86	6.5–7.3	13	0	13	15	1.9–330	13	0	24	7.4	5.0–208	14	0
	0^#^	0^#^	<LOQ^#^	0^#^	0^#^	13^#^	18^#^	9.9–272^#^	13^#^	0^#^	5^#^	6.9^#^	5.0–188^#^	5^#^	0^#^
*T-2	0	0	<LOQ	0	0	3	0.20	<LOQ -7.5	3	0	3	0.65	<LOQ-23	3	0
	0^#^	0^#^	<LOQ^#^	0^#^	0^#^	0^#^	0^#^	<LOQ^#^	0^#^	0^#^	0^#^	0^#^	<LOQ^#^	0^#^	0^#^
*ZEN	19	3.5	7.4–33	19	0	11	1.1	4.8–22	11	0	14	12	80–94	8	0
	0^#^	0^#^	<LOQ^#^	0^#^	0^#^	0^#^	0^#^	<LOQ^#^	0^#^	0^#^	5^#^	1.5^#^	<LOQ^#^	0^#^	0^#^
α-ZEL	31	31	<LOQ-140	6	0	5	6.5	<LOQ	0	0	0	0	<LOQ	0	0
	ND^#^	ND^#^	ND^#^	ND^#^	ND^#^	ND^#^	ND^#^	ND^#^	ND^#^	ND^#^	ND^#^	ND^#^	ND^#^	ND^#^	ND^#^
β-ZEL	19	4.7	<LOQ	0	0	8	2.9	<LOQ-63	3	0	0	0	<LOQ	0	0
	ND^#^	ND^#^	ND^#^	ND^#^	ND^#^	ND^#^	ND^#^	ND^#^	ND^#^	ND^#^	ND^#^	ND^#^	ND^#^	ND^#^	ND^#^
ΣZENs	44	39	72–155	31	0	11	10	<LOQ	0	0	14	12	80–94	8	0
	ND^#^	ND^#^	ND^#^	ND^#^	ND^#^	ND^#^	ND^#^	ND^#^	ND^#^	ND^#^	ND^#^	ND^#^	ND^#^	ND^#^	ND^#^

Key: ^#^ Parameter values for solvent extraction. % + ve Samples: percentage of positive samples (*i*.*e*. samples >LOD). Mean*: average contamination of the samples including positive (*i.e*. >LOD) and negative (*i.e*. <LOD) samples. Samples >Limit: number of samples with contamination levels above the maximum limit set by the EC. N.D.: not detected. AFB_1_: aflatoxin B_1_. AFB_2_: aflatoxin B_2_. AFG_1_: aflatoxin G_1_. AFG_2_: aflatoxin G_2_. AME: alternariol monomethyl ether. FB_1_: fumonisin B_1_. FB_2_: fumonisin B_2_. FB_3_: fumonisin B_3_. OTA: ochratoxin A. OTB: ochratoxin B. STEG: sterigmatocystin. T-2: T-2 toxin. ZEN: zearalenone. α-ZEL: α-zearalenol. β-ZEL: β-zearalenol. ΣZENs: sum of ZENs. ΣOTs: sum of OTs. ΣFB_1_&FB_2_: sum of FB_1_ and FB_2_. ΣAFs: sum of AFs. *Indicate the mycotoxins that are regulated by the EC (Arroyo-Manzanares *et al*., 2018. Commission Recommendation, 2013, 2006b), with their respective regulatory limits as follows: AFB_1_ in all cereals and their derived products 2 μg/kg and 4 μg/kg for ΣAFs^53^, ΣFB_1_&FB_2_ in unprocessed maize and maize flour/meal – 4,000 μg/kg (Arroyo-Manzanares *et al*., 2018. EC, 2007. WHO2017,A in unprocessed cereals – 5 μg/kg^53^, Sum of T-2 and HT-2 in unprocessed cereals – 100 μg/kg^121^, ZEN in unprocessed cereals other than maize – 100 μg/kg^45^ and ZEN in unprocessed maize – 350 μg/kg^45^.

#### Mycotoxin levels and incidence rate in maize, sorghum and millet from Nigeria

The incidence rates and levels of mycotoxin contamination in maize, sorghum and millet samples are presented in Table 5. All of the maize samples (n = 16), 32% (n = 38) of sorghum and 35% (n = 37) of millet samples were positive for at least one of the 15 tested mycotoxins. Fumonisins, in particular FB_1_ had the highest prevalence in terms of rate of occurrence and levels of contamination in all three cereals. All the maize samples (n = 16) were positive for FBs with contamination levels ranging from 17 to 7,947 µg/kg for FB_1_ and 30 to 14,603 µg/kg for ΣFB_1_&FB_2_. Out of the 16 analyzed maize samples, 7 samples (*i.e*., 44%) contained ΣFB_1_&FB_2_ at levels above the maximum levels of 4,000 µg/kg in unprocessed maize stipulated by the European Commission (EC) and CODEX^28,45,46^. Previous studies have equally reported high incidence rates and levels of FBs contamination in maize from Nigeria^20,47^. Bankole and Mabekoje^48^ found that FB_1_ was the predominant mycotoxin, occurring in 79% of samples of maize obtained from a similar region from Nigeria as we sampled (Southern Nigeria).

Such high incidence rates and even higher levels of FBs contamination have likewise been reported in other West African countries. Ngoko *et al*.^49^ found FB_1_ in 16 out of 18 maize samples from Cameroon at levels within the range 300 to 26,000 µg/kg. Fumonisins occurred in sorghum samples at relatively lower levels, with a mean value of 17 µg/kg and maximum value of 248 µg/kg for ΣFB_1_&FB_2_, while, no FBs contamination was recorded in millet samples. Chilaka *et al*.^12^ reported a mean value of 83 µg/kg and a maximum value of 180 µg/kg for ΣFB_1_&FB_2_ in sorghum. The International Agency for Research on Cancer (IARC) has classified FB_1_ as a group 2B carcinogen (possibly carcinogenic to humans)^50^. Consumption of foods contaminated with FBs have been directly linked with upper gastro-intestinal tract cancer^51^. Moreover, FBs are also nephrotoxic, hepatotoxic, immunosuppressive, atherogenic and embryotoxic in experimental animal systems^52^.

AFs contamination was also relatively high in the cereal samples. In maize, levels for AFB_1_ ranged from 6.5 to 315 µg/kg, with a mean of 54 µg/kg, while ΣAFs ranged from 16 to 323 µg/kg. Sixty-nine percent (n = 16) of the maize samples were contaminated above the maximum level of 2 and 4 µg/kg for AFB_1_ and ΣAFs, respectively, stipulated by the European Commission (EC)^28,53^. These levels are similar to those reported by Bandyopadhyay *et al*.^18^, who reported ΣAFs contamination ranging from 1.1 to 480 µg/kg and a mean of 36 µg/kg in freshly harvested maize in Nigeria. In sorghum, 8% of samples were above the EC limits for AFB_1_ (2 µg/kg) and ΣAFs (4 µg/kg), respectively, whereas, 5% of millet samples exceeded similar limits for AFB_1_ and ΣAFs. Aflatoxin B_2_, AFG_1_ and AFG_2_ were not detected in any of the millet samples, however, the observed levels for AFB_1_ (5.5 to 50 µg/kg) were in agreement to those reported by Apeh *et al*.^13^ (1.1 to 15 µg/kg) in millet grain from Nigeria. Observed levels for ΣAFs in sorghum (15 to 116 µg/kg) were less than those reported by Makun *et al*.^54^ (<LOQ to 1,164 μg/kg) in stored sorghum samples. This could be due to variations in fungi colonization of crops over different years which could be stimulated by annual variations in temperature and rainfall, in addition to other climatic conditions^55,56^. Generally, AFs were more prevalent in maize, followed by sorghum and then millet. A similar trend was observed by Bandyopadhyay *et al*.^18^, in their study on the relative severity of AFs contamination of cereal crops in West Africa. In fact, they observed that Nigerians consume 138 kg cereals annually, and if the main cereal is sorghum instead of maize, associated AF problems will be diminished 4-fold, whereas, if it is millet, then the AF-related risks will be reduced at least 8-fold^18^. Diversification of diets, instead of diets that are heavily dependent on maize could greatly reduce exposure to AFs and their consequent health-related problems. Aflatoxins are highly carcinogenic and are equally recognized as being immunosuppressive. Among the AFs group, AFB_1_ is considered the most toxic, and has been identified as the most potent naturally occurring carcinogen known to man^50,57^.

Ochratoxin A was present in 31% (n = 16) of the maize samples at levels ranging from 6.5 to 54 µg/kg. All positive maize samples for OTA were above the maximum level of 5 µg/kg in unprocessed cereals stipulated by the EC^53^. In sorghum, the levels of OTs varied from 7.5 to 15 µg/kg, while those for millet ranged from 0.75 to 20 µg/kg. Sangare-Tigori *et al*.^58^ reported higher OTA levels (17 to 204 μg/kg) in millet from the West African country of Côte d’Ivoire sampled between 1998 to 2002, which may be due to yearly variations in mycotoxin contamination patterns across the continent^55,56,59^. Exposure to OTA has been linked with nephropathy^60^, urinary tract tumors^61^ and oxidative DNA damage leading to mutagenesis and eventually cancer^62^. Based on its carcinogenicity in animal studies, OTA has also been classified as a group 2B possible human carcinogen by the International Agency for Research on Cancer (IARC)^63^.

The incidence rate and contamination range of ZEN in maize and sorghum and millet was 19% (7.4 to 33 µg/kg) and 11% (4.8 to 22 µg/kg), respectively. These levels are negligible when compared with the EC maximum limit of 350 μg/kg for unprocessed maize and 100 µg/kg for unprocessed cereals other than maize, respectively^45^. On the other hand, 5 out of 37 millet samples were positive for ZEN within the range 80 to 94 µg/kg, none of which exceeded the EC limits of 100 µg/kg in unprocessed cereals other than maize^45^. The ZEN analogues, α-ZEL and β-ZEL, were also detected in at least one of maize and sorghum samples each, while none of the analogues was detected in millet samples Chilaka *et al*. (2016) and Adetunji *et al*. (2014). reported maize and millet contamination with α-ZEL and β-ZEL^12,20^.

Cereal contamination by ZENs could be a major health concern as this toxin is known to be chemically stable both during various food processing operations such as cooking, heating, fermentation, milling etc., and has been quantified in a number of processed cereal-based products from Africa^64–66^. Zearalenone has been implicated in the manifestations of gynecomastia with testicular atrophy in rural males in Southern Africa^67^. Among human populations, children are the most vulnerable to ZEN exposure and the toxin has been implicated in several incidents of precocious pubertal changes^68^ and other fertility problems^69^. The potency of ZEN’s estrogenic activity is reportedly greater than that of many naturally occurring non-steroidal estrogens^70^. Exposure to high concentrations of ZEN in cattle feed has been linked with enlargement of the mammary gland, infertility, reduced milk production, vaginal secretions and vaginitis particularly in young dairy heifers^71^. Whereas in swine, effects of ZEN include enlargement of the uterus, vaginal prolapse, swelling of the vulva, infertility, reduced litre size and embryonic death^72^.

The Alternaria toxin, AME, and the trichothecene toxin, T-2, were not detected in any of the maize samples. Bankole *et al*.^73^ also reported the absence of T-2 contamination in maize from Nigeria. In sorghum (n = 38) and millet (n = 37) samples, T-2 occurred in 3% each, whereas, the average AME contamination was 0.99 and 21 µg/kg for sorghum and millet, respectively. Sterigmatocystin occurred in 13% of both maize (n = 16) and sorghum (n = 38) samples within the ranges of 6.5 to 7.3 µg/kg and 1.9 to 330 µg/kg, respectively, whereas 9 out of 37 millet samples were positive for STEG at concentrations ranging from 5 to 208 µg/kg. Elsewhere, STEG was reported as a contaminant of Nigerian maize^20^. While the toxicity of T-2 has been established in literature^74,75^, the toxic effects of STEG and AME to humans have remained largely limited. Nonetheless, it is known that STEG is a precursor for the biosynthesis of AFB_1_ and both have similar structural configurations, as such, STEG is considered as a potent mutagen, carcinogen, and teratogen^76,77^. The IARC classifies STEG as a group 2B carcinogen^77,78^.

A number of studies have demonstrated the stability of AME during extreme food processing conditions such as during wet baking of bread^66,79^, as such, there is a risk of secondary exposure to AME through processed cereal-based foods. Adekoya *et al*.^65^ reported AME contamination of gruels derived directly from maize and sorghum in Nigeria. Some studies have demonstrated the possible carcinogenicity and mutagenicity of AME^80–82^. For example, NIH/3T3 cells mutated by AME caused subcutaneous tumors in mice^83^. It has also been shown to induce DNA strand breaks in cell cultures^84^. In general, mycotoxin contamination was higher in maize, followed by sorghum and then millet (Table 5). A similar trend was observed by Makun *et al*.^85^ and Gwary *et al*.^86^. It has been shown that tannin-rich varieties of sorghum and millet are less susceptible to fungal colonization^13,87^, indicating that phytochemicals present in these cereals could exert antimycological properties as such resulting in less mycotoxin contamination.

#### Mycotoxin distribution patterns in maize, sorghum and millet from different agroecological zones of Nigeria

Geoclimatic conditions under which crops are cultivated are critical for fungi proliferation and attendant mycotoxin production^13,88,89^. Table 6 presents the distribution patterns of mycotoxins in maize, sorghum and millet across 5 agroecological zones in Nigeria. Maize samples generally had higher incidence rate and mycotoxin contamination levels. This could be because all the maize samples were obtained from the HRF/DRS agroecological zones. Also, as stated earlier in Section 3.2.1, maize is generally more susceptible to contamination by mycotoxigenic fungal species and consequent mycotoxin production as compared to sorghum and millet^12,90^. Likewise, in millet samples contamination levels increased from Southern to Northern Nigeria, with samples from Northern having the lowest levels of contamination and incidence rates. A similar trend was observed by Chilaka *et al*.^12^, who reported contamination/incidence rate of multi-mycotoxin contamination in DRS > NGS > SHS.

**Table 6:** Distribution of mycotoxins in maize, sorghum and millet across different agroecological zones in Nigeria as analyzed using PHWE followed by HPLC-MS/MS.

Mycotoxin	Maize		Sorghum				Millet					
	HRF/DRS (n = 16)		HRF/DRS (n = 12)		SHS (n = 26)		HRF/DRS (n = 20)		NGS/SGS (n = 9)		SHS (n = 8)	
	Mean* (µg/kg)	Range (µg/kg)	Mean* (µg/kg)	Range (µg/kg)	Mean* (µg/kg)	Range (µg/kg)	Mean* (µg/kg)	Range (µg/kg)	Mean* (µg/kg)	Range (µg/kg)	Mean* (µg/kg)	Range (µg/kg)
AFB_1_	54.41	6.50–315.00	10.92	15.00–116.00	0.28	<LOQ-7.25	2.55	<LOQ-49.50	4.17	<LOQ-37.50	0.00	<LOQ
AFB_2_	1.30	0.31–7.80	0.00	<LOQ	0.00	<LOQ	0.00	<LOQ	0.00	<LOQ	0.00	<LOQ
AFG_1_	0.00	<LOQ	0.00	<LOQ	0.28	<LOQ-7.25	0.00	<LOQ	0.00	<LOQ	0.00	<LOQ
AFG_2_	0.00	<LOQ	0.00	<LOQ	0.00	<LOQ	0.00	<LOQ	0.00	<LOQ	0.00	<LOQ
ΣAFs	55.71	15.60–322.80	10.92	15.00–116.00	0.56	<LOQ-14.50	2.55	<LOQ-49.50	4.17	<LOQ-37.50	0.00	<LOQ
AME	0.00	<LOQ	0.00	<LOQ	1.44	<LOQ-37.50	24.30	127.50–343.00	30.50	<LOQ-274.50	0.00	<LOQ
FB_1_	2,033.00	16.92–7,947.00	0.00	<LOQ	24.25	187.50–248.00	0.00	<LOQ	0.00	<LOQ	0.00	<LOQ
FB_2_	1,525.00	36.56–5,961.00	0.00	<LOQ	0.00	<LOQ	0.00	<LOQ	0.00	<LOQ	0.00	<LOQ
FB_3_	292.10	7.50–767.30	0.00	<LOQ	0.60	15.50–15.50	0.00	<LOQ	0.00	<LOQ	0.00	<LOQ
ΣFB_1_&FB_2_	3,851.00	29.60–14,603.00	0.00	<LOQ	24.25	187.50–248.00	0.00	<LOQ	0.00	<LOQ	0.00	<LOQ
OTA	8.28	6.50–54.25	1.88	7.50–15.00	0.00	<LOQ	1.36	6.88–20.25	0.00	<LOQ	0.00	<LOQ
OTB	1.52	7.75–16.50	0.63	<LOQ-7.50	0.00	<LOQ	0.68	6.75–6.88	0.00	<LOQ	0.00	<LOQ
ΣOTs	9.80	6.50–57.75	2.50	7.50–22.50	0.00	<LOQ	2.04	13.75–27.00	0.00	<LOQ	0.00	<LOQ
STEG	0.86	6.50–7.25	27.81	3.75–330.00	9.64	1.94–204.80	12.81	11.00–207.70	1.74	<LOQ -5.00	0.00	<LOQ
T-2	0.00	<LOQ	0.00	<LOQ	0.29	<LOQ-7.50	0.04	<LOQ	2.58	<LOQ-23.25	0.00	<LOQ
ZEN	3.49	7.38–33.00	3.06	7.50–21.75	0.18	4.75–4.75	9.99	90.00–90.00	26.94	80.00–93.50	0.94	<LOQ
α-ZEL	30.53	139.50–139.50	0.00	<LOQ	9.19	<LOQ	0.00	<LOQ	0.00	<LOQ	0.00	<LOQ
β-ZEL	4.66	<LOQ	5.25	63.00–63.00	1.80	<LOQ	0.00	<LOQ	0.89	<LOQ	0.00	<LOQ
ΣZENs	38.68	71.50–155.00	8.31	<LOQ	11.18	<LOQ	9.99	<LOQ-90.00	27.83	80.00–93.50	0.94	<LOQ

Key: HRF: Humid rain forest. DRS: Derived savannah. NGS: Northern guinea savannah. SGS: Southern guinea savannah. SHS: Sahel savannah. Mean*: average contamination of the samples including positive (i.e. >LOD) and negative (i.e. <LOD) samples. AFB_1_: aflatoxin B_1_. AFB_2_: aflatoxin B_2_. AFG_1_: aflatoxin G_1_. AFG_2_: aflatoxin G_2_. AME: alternariol monomethyl ether. FB_1_: fumonisin B_1_. FB_2_: fumonisin B_2_. FB_3_: fumonisin B_3_. OTA: ochratoxin A. OTB: ochratoxin B. STEG: sterigmatocystin. T-2: T-2 toxin. ZEN: zearalenone. α-ZEL: α-zearalenol. β-ZEL: β-zearalenol. ΣZENs: sum of ZENs. ΣOTs: sum of OTs. ΣFB_1_&FB_2_: sum of FB_1_ and FB_2_. ΣAFs: sum of AFs.

The observed high incidence rate and contamination levels of mycotoxins in maize, sorghum and millet samples from the HRF/DRS agroecological zones could be partly due to the climatic conditions in these regions that favor the proliferation of mycotoxigenic fungi species and subsequent mycotoxin production^22^. As already described in Section 2.2.1, the HRS is characterized by abundant rainfall (1,500 to 2,000 mm/yr), high humidity (78 to 100%) and average temperatures between 25 to 28 °C^91,92^, while the DRS vegetation represents a transition between the humid rainforest and guinea savannah zones. Annual rainfall in this zone ranges from 1,200 mm to 1,700 mm, while average humidity and temperature are 66 to 78% and 26 to 27 °C, respectively^93^. Adejumo and Adejoro^93^, noted that the important agroecological zones in terms of mycotoxin research in Nigeria are the HRF, DRS and SGS/NGS zones.

Interestingly, this HRF zone spans across many of the West African countries, as such, similar patterns of mycotoxin contamination have been recorded in some of these countries^49,94^. Hell *et al*.^94^, Udoh *et al*.^95^ and Gong *et al*.^96^ observed significant AFs contamination of maize in lowland areas in Cameroon, Nigeria, Benin and Togo, respectively. A similar trend was reported for FBs (10 to 16,040 μg/kg) in maize from Burkina Faso^97^ and FB_1_ (300 to 26,000 μg/kg) in maize from Cameroon^49^. On the other hand, the lower levels of mycotoxin contamination and incidence rates observed in the Middle-Belt and Northern parts of Nigeria *i.e*., the SGS/NGS and SHS, respectively, could be due to dryer and more arid climatic conditions in these regions. Particularly the SHS has a much lower annual rain fall and humidity as compared to DRS and HRF. Temperatures can reach as high as 40 °C^22,98^. Such conditions may not favor the proliferation of mycotoxigenic fungi species.

#### Simultaneous occurrence of multiple mycotoxins in maize, sorghum and millet from Nigeria as analyzed using PHWE followed by HPLC-MS/MS

Based on the results of this study, exposure of humans and animals to multiple mycotoxins is highly likely in the selected regions in Nigeria, as we observed co-occurrence of different groups of mycotoxins in many of the analyzed samples. This observation is in line with previous literature reports^12,99,100^. Mycotoxin co-contamination of crops is a complex phenomenon, and a number of interrelating factors, such as fungi species, crop genotype, and climatic conditions may be responsible for the co-occurrence of mycotoxins in foods. It is known that a single fungal species may be able to produce more than one mycotoxin^101,102^, while one mycotoxin can be produced by different fungi species^103,104^.

Table 7 and Appendix B describe all the possible co-occurrence patterns of the five groups of regulated mycotoxins detected in the samples, which are AFs, FBs, OTs, T-2 and ZENs. This was achieved using the Venn diagram web application (Bioinformatics and Evolutionary Genomics, Ghent University, Belgium)^105^. The 5 groups of mycotoxins subjected to this analysis yielded 9 different unique intersections as summarized in Table 7. The highest co-occurrence of mycotoxins that appeared in the maize samples was the co-contamination of AFs and FBs (*i*.*e*. AFs + FBs) which occurred in 6 (n = 16) of the maize samples, while 3 of the samples simultaneously contained AFs, FBs and OTs. A similar pattern was observed in 5,000 samples submitted to the GEMS/Food contaminants database between 2011 and 2016, where reiterations of FBs + AFs combinations occurred in approximately 6% of the maize samples, 1% of sorghum^106^. A combination of AFs + FBs + OTs + ZENs also occurred in one (n = 16) of the maize samples, whereas, a combination of AFs + OTs + ZENs occurred in one (n = 38) of the sorghum samples. The co-occurrence of OTs + ZENs was observed in a single millet sample.

**Table 7:** Co-occurrence of different groups of regulated mycotoxins in maize, sorghum and millet samples from Nigeria.

Co-contaminants	Number of co-occurrences		
	Maize (n = 16)	Sorghum (n = 38)	Millet (n = 37)
AFs + FBs	6	0	0
AFs + FBs + OTs	3	0	0
AFs + FBs + OTs + ZENs	1	0	0
AFs + FBs + ZENs	1	0	0
AFs + OTs + ZENs	0	1	0
AFs + ZENs	0	1	0
FBs + OTs + ZENs	1	0	0
FBs + T-2	0	1	0
OTs + ZENs	0	0	1

Key: AFs: aflatoxins. FBs: fumonisins. OTs: ochratoxins. T-2: T-2 toxin. ZENs: zearalenone and its analogues α- and β-zearalenol.

Such co-contamination patterns have previously been reported in cereals from Nigeria^12,20,48,107^. Bankole and Mabekoje^48^ reported that 15 samples (n = 103) of pre-harvest maize from Southern Nigeria were contaminated with both FBs and AFs simultaneously. A prevalence rate of 10% and mean contamination level of 111 µg/kg have been reported for OTA, concurrently with OTB 7% (7.5 µg/kg), STEG 37% (3 µg/kg), ZEN 17% (174 µg/kg), α-ZEL 1% (17 µg/kg), and β-ZEL 1% (13 µg/kg) in stored maize from five different agroecological zones in Nigeria^20^. A review of over a hundred papers between 1987 to 2016, revealed 127 mycotoxin combinations, of which AFs + FBs, AFs + OTA, DON + ZEN, and FBs + ZEN were amongst the most frequently co-occurring combinations in cereal crops^108^. In Tanzania, co-exposure to FBs + AFs has been confirmed by means of plasma or urinary biomarkers of AF_1_ and FB_1_^106^. Co-occurrence can be caused by substrate colonization with a single fungus that produces more than one mycotoxin, or due to colonization by different fungi species that produce different mycotoxins. It has been reported that ZEN usually co-occurs with one or more of the trichothecenes (THs), because of the ability of its producing fungi to synthesize more than one mycotoxin^109^.

Since the individual toxins that make these combinations are all amongst the most potent mycotoxins, their co-existence must not be neglected^108^. The combined effects of different mycotoxins have been extensively reviewed in literature^106,108,110^, and could manifest as additive, synergistic or antagonistic^106^. For example, exposure of F344 rats to FB_1_ + AFB_1_ combinations increased liver preneoplastic changes suggestive of a synergistic interaction^111^, whereas, health concerns in humans include possible childhood stunting^112^. All cytotoxic effects of the binary combinations of OTA, FB_1_ and AFB_1_ in low concentrations at their EU regulatory limits to MDBK cell lines were additive, and in the order OTA + FB_1_ > AFB_1_ + FB_1_ > AFB_1_ + OTA^113^, while FB_1_ + α-ZEL combination significantly diminished interferon γ mRNA expression as compared to α-ZEL alone^114^.

### Significance of mycotoxin contamination of Nigerian staple cereals

We reiterate that mycotoxin contamination of maize, sorghum and millet crops in Nigeria represents a major food safety concern because these crops are staples. Atanda *et al*.^14^ asserted that AF contamination in maize and peanuts in Nigeria contributed to at least 7,761 cases of liver cancer, resulting in a total burden of 100,965 Disability-Adjusted Life Years (DALYs)^14^. Derived food products from these cereals such as *masa*, *tuwo*, *ogi*, *ogi-baba*, *kunu*, *burukutu* and others are also at risk of mycotoxin contamination^12,65,93,115–118^. Some of these processed food products (e.g. *ogi* and *ogi*-*baba*) are used as weaning foods for children. Sadly, a number of studies have reported mycotoxin contamination in these weaning foods^65,119,120^. In fact, a study^120^, reported AFB_1_ contamination levels up to 4,806 µg/kg in home-made weaning food made from maize and soybeans^120^. Moreover, a posthumous autopsy of infants who suffered from kwashiorkor showed a significant level of AFs in their brains, because of consumption of contaminated maize-derived gruels^15^.

Aside the health effects on humans and animals, mycotoxin prevalence in Nigeria and Africa as a whole has other significant socio-economic impacts, ranging from food security, decreased market value of crops, regulatory rejections of goods mainly at ports of exit, damage to the African agricultural export brand amongst others^5^. For example, in 2010, the monetized burden of AFs contamination in Nigeria was estimated to be between 112 million U$D and 942 million U$D, which accounts for roughly 0.5% of the nation’s Gross Domestic Product (GDP)^14^. These enormous impacts of mycotoxin could significantly jeopardize prospects of attaining the UN’s sustainable development goal number 2, of achieving food security, improved nutrition, and a healthy agroeconomic growth by 2030^5^.

Based on the results obtained in this study and considering the economic importance of these cereals in Nigeria, it is thus imperative to prioritize the adoption of functional mitigation strategies that are both cost-effective, crop-specific and locally adapted to the climatic conditions and agronomic practices of the region, in order to adequately combat the prevalence of these toxins in Nigeria. Further to this, more research should be done to determine the toxicological effects of different mycotoxin combinations in order to better understand their associated public health risks. If this is done, then future establishment of regulatory limits or as the case may be, reassessment of already existing limits could take into account the effects of co-existing mycotoxins. The successful adoption of PHWE in this study further represents a step closer to sustainability in green-solvent extraction in the field of mycotoxicology.

## Supplementary information

Supplementary information.

Supplementary information 2
