# Supplementary material for: Parallel validation of a green-solvent extraction method and quantitative estimation of multi-mycotoxins in staple cereals using LC-MS/MS
Source: Sci Rep. 2020 Jun 25;10:10334. doi: 10.1038/s41598-020-66787-z (PMC7316717; doi:10.1038/s41598-020-66787-z)
Supplement: Supplementary file 1 — Supplementary information. [file 41598_2020_66787_MOESM1_ESM.docx]

## Parallel validation of a green-solvent extraction method and quantitative estimation of multi-mycotoxins in staple cereals using LC-MS/MS

Sefater Gbashi^1^*, Patrick Berka Njobeh^1^*, Ntakadzeni Edwin Madala^2^, Marthe De Boevre^3^, Victor Kagot^3^, Sarah De Saeger^1,3^

^1^Department of Biotechnology and Food Technology, Faculty of Science, University of Johannesburg, P.O Box 17011, Doornfontein Campus, 2028, Gauteng, South Africa.

^2^Department of Biochemistry, School of Mathematical and Natural Sciences, University of Venda, Thohoyandou, South Africa.

^3^Centre of Excellence in Mycotoxicology and Public Health, Department of Bioanalysis, Ghent University, 9000 Ghent, Belgium.

***Corresponding authors**: Njobeh, P. B.; **Tel:** +27 11 559 6803; **Fax:** +27 11 559 6651; **Email:** [pnjobeh@uj.ac.za](mailto:pnjobeh@uj.ac.za); Gbashi, S.; **Email:** [sefatergbashi@gmail.com](mailto:sefatergbashi@gmail.com)

**Appendix A:** Linearity parameters and matrix effect of PHWE of multi-mycotoxins in maize, sorghum and millet.

| **Myco** |  | **Maize** | | | **Sorghum** | | | **Millet** | | |
| --- | --- | --- | --- | --- | --- | --- | --- | --- | --- | --- |
|  | **Linear Range (µg/kg)** | **Equation** | ***R^2^*** | **ME (%)** | **Equation** | ***R^2^*** | **ME (%)** | **Equation** | ***R^2^*** | **ME (%)** |
| **AFB_1_** | 30-1,000 | y=726418.00x+0.0000000 | 0.998 | 43 | y=758813.00x+0.0000000 | 0.998 | 45 | y=394509.00x+0.0000000 | 0.998 | -5 |
|  | 10-40^#^ | y=0.0388236x-0.0297569^#^ | 0.999^#^ | ND^#^ | y=0.02246.00x-0.0551951^#^ | 0.992^#^ | ND^#^ | y= 0.0205976x-0.0481812^#^ | 0.994^#^ | ND^#^ |
| **AFB_2_** | 10-300 | y=29922.300x+0.0000000 | 0.990 | ND | y=30003.100x+0.0000000 | 0.996 | ND | y=1017810.0x+0.0000000 | 0.999 | -14 |
|  | 10-40^#^ | y= 0.0389391x-0.0724287^#^ | 0.999^#^ | ND^#^ | y=0.0217079x-0.0288360^#^ | 0.972^#^ | ND^#^ | y= 0.0182912x-0.0454976^#^ | 0.988^#^ | ND^#^ |
| **AFG_1_** | 30-1,000 | y=733883.00x+0.0000000 | 0.997 | 49 | y=656177.00x+0.0000000 | 0.984 | 43 | y=364241.00x+0.0000000 | 0.991 | -3 |
|  | 10-40^#^ | y= 0.0282378x-0.0290143^#^ | 0.999^#^ | ND^#^ | y= 0.0190237x-0.0468256^#^ | 0.988^#^ | ND^#^ | y= 0.0155844x-0.0182782^#^ | 0.993^#^ | ND^#^ |
| **AFG_2_** | 30-1,000 | y=602178.00x+0.0000000 | 0.999 | 44 | y=596522.00x+0.0000000 | 0.995 | 44 | y=358975.00x+0.0000000 | 0.998 | 6 |
|  | 10-40^#^ | y=0.0137443x -0.0346290^#^ | 0.999^#^ | ND^#^ | y= 0.0197771x-0.0440019^#^ | 0.984^#^ | ND^#^ | y= 0.0117548x-0.0147432^#^ | 0.982^#^ | ND^#^ |
| **AME** | 30-1,000 | y=282992.00x+0.0000000 | 0.998 | 57 | y=269502.00x+0.0000000 | 0.994 | 55 | y=134284.00x+0.0000000 | 1.000 | 9 |
|  | 10-40^#^ | y=0.0012392x-0.0053106^#^ | 0.999^#^ | ND^#^ | y=0.0006475x-0.0170763^#^ | 0.996^#^ | ND^#^ | y=0.0005798x-0.0085391^#^ | 0.989^#^ | ND^#^ |
| **FB_1_** | 60-2,000 | y=108498.00x+0.0000000 | 0.992 | 63 | y=109.64500x+0.0000000 | 0.998 | 64 | y=56694.100x+0.0000000 | 0.998 | 30 |
|  | 20-80^#^ | y=0.0031071x-0.0163114^#^ | 0.998^#^ | ND^#^ | y=0.0021193x-0.0772540^#^ | 0.989^#^ | ND^#^ | y= 0.0014962x-0.0995423^#^ | 0.984^#^ | ND^#^ |
| **FB_2_** | 16-500 | y=15175200x+0.0000000 | 0.997 | 57 | y=16315600x+0.0000000 | 0.996 | 60 | y=9292170.0x+0.0000000 | 0.995 | 30 |
|  | 20-80^#^ | y=0.0036764x+0.0021624^#^ | 0.999^#^ | ND^#^ | y=0.00188148x-0.0115376^#^ | 0.989^#^ | ND^#^ | y=0.0015081x-0.1082910^#^ | 0.977^#^ | ND^#^ |
| **FB_3_** | 30-1,000 | y=467233.00x+0.0000000 | 0.997 | 61 | y=494279.00x+0.0000000 | 0.999 | 63 | y=242114.00x+0.0000000 | 0.998 | 24 |
|  | 20-80^#^ | y= 0.0051078x-0.0189341^#^ | 0.999^#^ | ND^#^ | y= 0.0036043x-0.0766144^#^ | 0.987^#^ | ND^#^ | y=0.0023971x-0.1025880^#^ | 0.974^#^ | ND^#^ |
| **OTA** | 30-1,000 | y=2569440.0x+0.0000000 | 0.999 | 60 | y=2462030.0x+0.0000000 | 0.998 | 59 | y=1242000.0x+0.0000000 | 0.999 | 18 |
|  | 25-100^#^ | y= 0.0059043x-0.0187238^#^ | 0.999^#^ | ND^#^ | y=0.0028299x-0.0278753^#^ | 0.991^#^ | ND^#^ | y= 0.0042255x-0.0060401^#^ | 0.968^#^ | ND^#^ |
| **OTB** | 30-1,000 | y=25504200x+0.0000000 | 0.996 | 62 | y=21534600x+0.0000000 | 0.997 | 55 | y=1165260.0x+0.0000000 | 1.000 | 18 |
|  | N.D. ^#^ | N.D. ^#^ | N.D. ^#^ | N.D. ^#^ | N.D. ^#^ | N.D. ^#^ | N.D. ^#^ | N.D. ^#^ | N.D. ^#^ | N.D. ^#^ |
| **STEG** | 30-1,000 | y=1697490.0x+0.0000000 | 0.995 | 60 | y=1766930.0x+0.0000000 | 0.997 | 61 | y=794631.00x+0.0000000 | 0.999 | 14 |
|  | 25-100^#^ | y=0.0189498x-0.0007388^#^ | 0.999^#^ | ND^#^ | y= 0.0087249x-0.0812727^#^ | 0.988^#^ | ND^#^ | y=0.0097481x-0.0207013^#^ | 0.999^#^ | ND^#^ |
| **T-2** | 30-1,000 | y=15881.200x+0.0000000 | 0.996 | 61 | y=62117.600x+0.0000000 | 0.998 | 90 | y=33360.200x+0.0000000 | 0.999 | 82 |
|  | 50-200^#^ | y=0.00739964x-0.0429551^#^ | 0.999^#^ | ND^#^ | y=0.0033890x-0.0488755^#^ | 0.993^#^ | ND^#^ | y= 0.0038011x+0.0089581^#^ | 0.987^#^ | ND^#^ |
| **ZEN** | 30-1,000 | y=571027.00x+0.0000000 | 0.997 | 60 | y=494014.00x+0.0000000 | 0.997 | 54 | y=240378.00x+0.0000000 | 0.993 | 6 |
|  | 50-200^#^ | y=0.0010048x-0.0007694^#^ | 0.996^#^ | ND^#^ | y=0.0002811x-0.0015622^#^ | 0.917^#^ | ND^#^ | y=0.0002970x-0.0043136^#^ | 0.969^#^ | ND^#^ |
| **α-ZEL** | 30-1,000 | y=48991.100x+0.0000000 | 0.999 | 61 | y=45737.600x+0.0000000 | 0.999 | 58 | y=20146.000x+0.0000000 | 0.998 | 5 |
|  | N.D. ^#^ | N.D. ^#^ | N.D. ^#^ | N.D. ^#^ | N.D. ^#^ | N.D. ^#^ | N.D. ^#^ | N.D. ^#^ | N.D. ^#^ | N.D. ^#^ |
| **β-ZEL** | 30-1,000 | y=128721.00x+0.0000000 | 0.999 | 60 | y=257624.00x+0.0000000 | 0.997 | 56 | y=128721.00x+0.0000000 | 0.999 | 13 |
|  | N.D. ^#^ | N.D. ^#^ | N.D. ^#^ | N.D. ^#^ | N.D. ^#^ | N.D. ^#^ | N.D. ^#^ | N.D. ^#^ | N.D. ^#^ | N.D. ^#^ |
| **Mean**^#^ |  |  | 0.996±0.00 |  |  | 0.996±0.00 |  |  | 0.996±0.00 |  |
|  |  |  | 0.999±0.00^#^ |  |  | 0.982±0.01^#^ |  |  | 0.999±0.00^#^ |  |
| **Sign (2-tailed)** |  |  | 0.01 |  |  | 0.04 |  |  | 0.01 |  |

Key: ^#^ Parameter values for solvent extraction. Myco: mycotoxin. ME: Matrix effect. N.D.: not detected. *R^2^*: coefficient of determination. AFB_1_: aflatoxin B_1_. AFB_2_: aflatoxin B_2_. AFG_1_: aflatoxin G_1_. AFG_2_: aflatoxin G_2_. AME: alternariol monomethyl ether. FB_1_: fumonisin B_1_. FB_2_: fumonisin B_2_. FB_3_: fumonisin B_3_. OTA: ochratoxin A. OTB: ochratoxin B. STEG: sterigmatocystin. T-2: T-2 toxin. ZEN: zearalenone. α-ZEL: α-zearalenol. β-ZEL: β-zearalenol.
