# Supplementary material for: Parallel validation of a green-solvent extraction method and quantitative estimation of multi-mycotoxins in staple cereals using LC-MS/MS
Source: Sci Rep. 2020 Jun 25;10:10334. doi: 10.1038/s41598-020-66787-z (PMC7316717; doi:10.1038/s41598-020-66787-z)
Supplement: Supplementary file 2 — Supplementary information 2 [file 41598_2020_66787_MOESM2_ESM.docx]

## Parallel validation of a green-solvent extraction method and quantitative estimation of multi-mycotoxins in staple cereals using LC-MS/MS

Sefater Gbashi^1^*, Patrick Berka Njobeh^1^*, Ntakadzeni Edwin Madala^2^, Marthe De Boevre^3^, Victor Kagot^3^, Sarah De Saeger^1,3^

^1^Department of Biotechnology and Food Technology, Faculty of Science, University of Johannesburg, P.O Box 17011, Doornfontein Campus, 2028, Gauteng, South Africa.

^2^Department of Biochemistry, School of Mathematical and Natural Sciences, University of Venda, Thohoyandou, South Africa.

^3^Centre of Excellence in Mycotoxicology and Public Health, Department of Bioanalysis, Ghent University, 9000 Ghent, Belgium.

***Corresponding authors**: Njobeh, P. B.; **Tel:** +27 11 559 6803; **Fax:** +27 11 559 6651; **Email:** [pnjobeh@uj.ac.za](mailto:pnjobeh@uj.ac.za); Gbashi, S.; **Email:** [sefatergbashi@gmail.com](mailto:sefatergbashi@gmail.com)


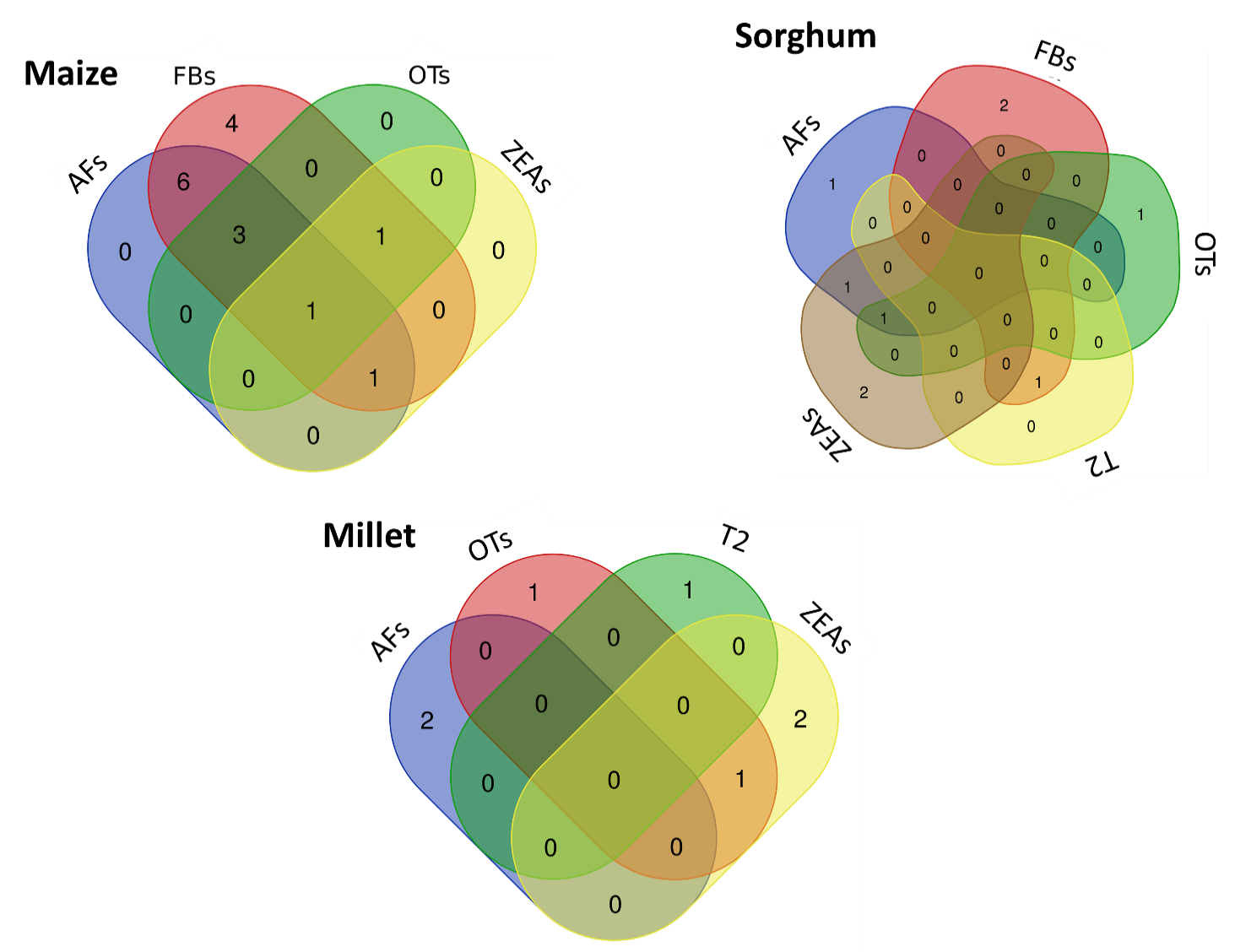


**Appendix B:** Incidences of co-contamination of different classes of regulated mycotoxins in maize, sorghum and millet from Nigeria as analyzed using PHWE followed by HPLC-MS/MS. AFs: aflatoxins. FBs: fumonisins. OTs: ochratoxins. T-2: T-2 toxin. ZENs: zearalenone and its analogues α- and β-zearalenol.
